# Mechanical Properties of Cellulose and Flax Fiber Unidirectional Reinforced Plywood

**DOI:** 10.3390/polym14040843

**Published:** 2022-02-21

**Authors:** Johannes Jorda, Günther Kain, Marius-Catalin Barbu, Berndt Köll, Alexander Petutschnigg, Pavel Král

**Affiliations:** 1 Forest Products Technology and Timber Construction Department, Salzburg University of Applied Sciences, Markt 136a, 5431 Kuchl, Austria; johannes.jorda@fh-salzburg.ac.at (J.J.); marius.barbu@fh-salzburg.ac.at (M.-C.B.); alexander.petutschnigg@fh-salzburg.ac.at (A.P.); 2 Department for Furniture and Interior Design, Higher Technical College Hallstatt, Lahnstraße 69, 4830 Hallstatt, Austria; 3 Faculty for Furniture Design and Wood Engineering, Transilvania University of Brasov, B-dul. Eroilor Nr. 29, 500036 Brasov, Romania; 4 Lenzing Aktiengesellschaft, Werkstrasse 2, 4860 Lenzing, Austria; b.koell@lenzing.com; 5 Department of Wood Science and Technology, Mendel University, Zemĕdĕlská 3, 61300 Brno, Czech Republic; kral@mendelu.cz

**Keywords:** plywood, natural fiber-reinforcement, cellulose, flax

## Abstract

This research presents the influence of two different cellulose (hydrophobic pretreated/non-pretreated) and one flax-fiber unidirectional nonwoven low areal weight fiber reinforcements on the mechanical properties of urea-formaldehyde bonded five layered beech (*Fagus sylvatica* L.) plywood as an alternative to commonly used synthetic fiber reinforcements. The results display divergent trends regarding the improvement of the mechanical properties—modulus of elasticity, modulus of rupture, tensile strength, shear strength, and screw withdrawal resistance. The non-treated cellulose and flax reinforcing nonwoven fabrics revealed similar mechanical behaviors. The hydrophobic pretreatment of cellulose nonwovens improved the performance of plywood regarding tensile strength (10–11%), shear strength (7–16%), screw withdrawal resistance (11–15%), and modulus of rupture (0–2%), but lowered modulus of elasticity (2–3%) compared to the reference.

## 1. Introduction

Plywood, one of the oldest continually used wood-based materials in the history of mankind [1,2], is considered in late maturity or decline stage of its product life cycle [3] with an expected global annual growth rate of 2.23% to 7.8% for the coming years [4,5]. Despite the negative forecast of the last decades, plywood is by far the most produced wood-based panel with an annual global production of 160 to 180 million m^3^ (2018) [6]. Beside economic aspects, it increasingly attracts research interest [7]. According to standard EN 313-2 plywood is defined “as a wood-based panel consisting of an assembly of layers glued together with the direction of the grain in adjacent layers usually at right angles”. The plies consist of thin sheets of wood with a thickness less than 7 mm [8]. Plywood utilizes the concept of laminar structured composite materials [9] and the concept of structural dissolution of wood inherent to wood-based products [10]. Structural dissolution, introduced by Marra in 1972, describes the different stages of downsizing round wood to fibers or chemical constituents such as cellulose [10,11]. Fibers are technically defined as an elongated object with a length-to-diameter ratio greater than one [9]. Fibers can be embedded continuously or discontinuously within a matrix, wherein they can be oriented or randomly aligned resulting in high-performance engineering materials [12]. High quality fibers are characterized by small diameter, high flexibility, and high length-to-diameter ratio [13]. Wood itself can be defined as a natural, polymeric, cellular fiber composite [2].

Fibers used for reinforcing fabrics can be divided into two main classes of origin: synthetic and natural fibers [14]. Natural fiber reinforced materials are proposed as a new material class and referred to as natural fiber composites (NFCs). These are characterized by lightness and reduced environmental impact [15]. Natural fiber composites are subdivided by the origin of fibers into plant-, animal-, and mineral-based fibers [14]. Research on reinforcing wood-based products with primarily synthetic fiber-based fabrics to enhance mechanical properties date back to the 1960s [16]. The latest research regarding natural fiber reinforcement of plywood focuses strongly on the use of mineral-based basalt fiber [17,18,19,20]. Whereas the application of the fiber in wood-based composites is normally located at the surface due to the improvement for tensile strength compared to native wood under load barring induced static bending [17].

Reinforcing fabrics, such as glass, carbon or basalt, located at the surface of wood-based panels do have several severe drawbacks: first the high energy consumption during production, second the necessity of special woodworking tools for postproduction and third the limitation of applicable surface treatments narrowing the range of applications. Lignocellulosic fiber fabrics like flax or cellulose can be applied as reinforcement to overcome these drawbacks. Several promising studies about flax fiber reinforcement for laminar wood structures have been carried out during the last decade [21,22,23,24,25,26,27].

Flax (*Linum usitatissimum* L.) is considered as one of the strongest plant fibers competitive to E-glass [28]. Based on its lignocellulosic origin, the polymer constituents are cellulose (~75%), hemicellulose (~20%), lignin (~4%), pectin (~4%), and waxes and fats (~0.7%) [29]. The fiber length is in the range of 20 to 50 mm, the diameter between 15 and 20 μm, the wall thickness ranges from 4 to 12 μm, and the microfibril angle is as low as 5° [30]. The modulus of elasticity (MOE) accounts for 44 kN/mm² (E_fL1_ = 57 kN/mm^2^, respectively for *E*_fL2_ 44.5 kN/mm² [28]) and the tensile strength (TS) for 745 N/mm² [31]. The high state of research, the low ecological impact during production and the industrial availability makes flax suitable as a competitive bio-based ecological-friendly reinforcing material [15].

Cellulose, a polysaccharide polymer, is the supportive structure of plants with an abundant availability [32]. It is considered to be one of the gamechangers for developing the bioeconomy mandatory to handle declining fossil and mineral resources as well as climate change [30]. Research for possible applications has a great variety, ranging from textiles and biomedicine to energy storage. Regarding the mechanical properties of cellulose, there is a difference between different possible molecular structures of cellulose. The elastic modulus of amorph structured cellulose I (native cellulose) is twice as high as the one of cellulose II (NaOH treated cellulose I) used for regenerated cellulose products (Modal, Lyocell, Viscose) [33,34]. Adusumali et al. (2006) investigated the mechanical properties of different regenerated cellulose fibers (Lyocell MOE *E* 23.4–30.5 kN/mm², TS 556–790 N/mm²) in comparison to flax and glass fiber. The results displayed, including the effect of lower density of cellulose (1.5 g/m³), significant lower values of modulus of elasticity and tensile strength (TS) for regenerated cellulose compared to flax and glass. In contrast, the high failure strain of regenerated cellulose fibers is suitable for composite applications with high fracture toughness [35]. The modulus of elasticity of regenerated cellulose is twice the one of wood and wood-based products such as massive beech (*Fagus sylvatica* L.) with a modulus of elasticity of 10 to 18 kN/mm² [36] or beech plywood with a modulus of elasticity of approximately 14 kN/mm² [37].

Concluding from previous studies that there should be a significant difference between the mechanical properties of plywood depending on the type of reinforcement. Further, the aspect of bonding performance for lignocellulose fiber-based reinforcement with regard to the fiber adhesive matrix is seen as a key-feature for exploiting the full potential of bio-based reinforcing materials.

The aim of the study is to investigate the influence of two different (hydrophobic/non-hydrophobic pretreated) unidirectional cellulose and one flax fiber fabric reinforcements within the glue line on the mechanical properties (modulus of elasticity, modulus of rupture, tensile strength, shear strength, internal bond, screw withdrawal resistance) of urea-formaldehyde bonded laminar structured plywood.

## 2. Materials and Methods

### 2.1. Materials and Sample Preparation

Rotary cut beech (*Fagus sylvatica* L.) pre-conditioned (20 °C, 65% relative air humidity) veneers (purchased by Europlac, Topolčany, Slovakia) with a nominal thickness of 2.2 mm, the dimensions 0.75 m × 0.75 m, an average density 0.72 g/cm³ and an average moisture content of 12% were used as wooden raw material. Two different unidirectional cellulose-based LENZINGTM Lyocell fabrics (Lenzing, Austria) and one unidirectional flax fabric Lineo FlaxTape 50 (distributed by Ecotechniln, Valliquerville, France) with a grammage of 50 g/m² acted as fiber reinforcement. The Lyocell fibers had a linear density of 1.7 dtex. The Lyocell fabrics with a grammage of 50 g/m^2^ (Variant Lyocell A and B) differed by their pretreatment. Variant A was non-treated, whereas variant B had a hydrophobic treatment. The Lineo Flaxtape (Variant C) had an estimated density of 1.31 g/cm³ and a thickness of 0.1 mm. The variants A, B, and C are defined by the type of reinforcing fabric (A = untreated Lyocell, B = hydrophobic pretreated Lyocell, and C = FLAXTape).

Urea-formaldehyde (UF) 1274 Akzo Nobel (Akzo Nobel, Stockholm, Sweden) and hardener 2545 Akzo Nobel (Akzo Nobel, Stockholm, Sweden) with a density of 1.3 and 1.45 g/cm³, respectively, a viscosity of 1.5 to 3.5 Pa·s/2.0 to 10.0 Pa·s and a resin hardener ratio of 100:20 g was used as adhesive.

**Figure 1:**
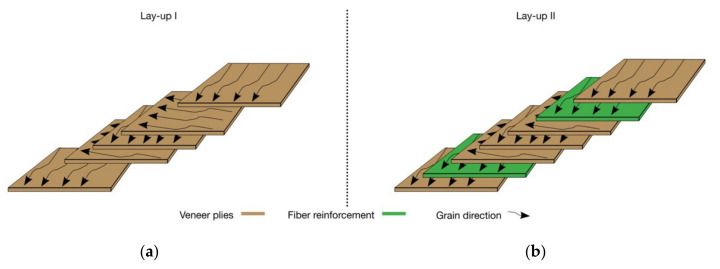
(**a**) Lay-up I for the reference. (**b**) Lay-up II for the reinforced variants A/A+, B/B+, and C/C+.

Two lay-ups of plywood were introduced. Lay-up I for the reference sample (Ref) consisted of five 90° cross-laid-veneer plies (Figure 1a). The lay-up II for the fiber reinforced plywood consisted of five 90° cross-laid-veneer plies with one layer of fiber reinforcing fabric located at each outer glue line of the specimen. The unidirectional fiber reinforcement was orientated according to the grain direction of the surface veneer (Figure 1b).

The adhesive amount of UF was set to 160 g/m² for wood-to-wood bonding and wood to fabric for the reference (Ref) and variant A, B, and C. For variant A+, B+, and C+ the adhesive amount was set to 160 g/m² for wood to wood and 200 g/m² for wood to fabric bonding (Table 1).

Plywood boards with the dimensions 0.75 m × 0.75 m and an average thickness of 10.0 mm were produced using a Höfler HLOP 280 press (Taiskirchen, Austria) with a press time of 12.75 min, a specific pressure of 3 N/mm² and a temperature of 110 °C. The lay-up and adhesive application were carried out manually. Adhesive application was controlled by weighing with a KERN ITB 35K1IP device (Baligen-Frommern, Germany).

The boards were stored until mass constancy in a constant climate of 20 °C and 65% relative humidity. Test specimen were cut from the plywood boards for the determination of density, bending strength (MOR), stiffness (MOE), tensile- (TS), shear strength (SS), internal bond (IB), and screw withdrawal resistance (SWR) (Table 1).

### 2.2. Testing

**Figure 2:**
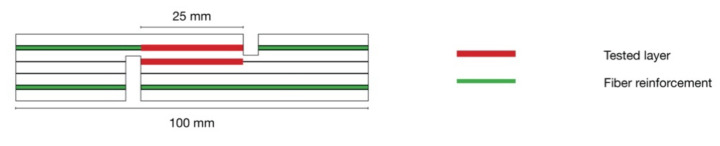
Shear test specimen dimensions of five layered unidirectional reinforced plywood according to EN 314.

The density was determined according to EN 323:2005 and obtained from the bending test specimen [38]. The density profile was measured with a DENSE-LAB X (EWS, Hammeln, Germany) and the specimen dimensions 50 × 50 mm. The density profile graph is based on the mean of the 5 tested specimens per variant. Thickness was obtained from the bending test specimen. The “Degree of compression” was calculated by the percentage-based difference between the theoretical thickness of 11 mm of non-compressed veneer ply stack before pressing and the actual thickness of the bending test specimen according to Spulle et al. (2021) [39]. The influence of fabric thickness is negligible due to its relative thickness of less than 1% of the theoretical thickness of the prepressed plywood stack. Modulus of rupture (MOR) and modulus of elasticity (MOE) were determined by a three-point bending test according to EN 310:2005 with specimen dimensions 250 × 50 mm [40]. The screw withdrawal resistance (SWR) was measured according to EN 320:2011 with the specimen dimensions of 50 × 50 mm and thread screws ST 4.2 mm [41]. The tensile strength (TS) was determined following DIN 52377 [42]. Shear strength (SS) was determined according to EN 314:2005 with specimen dimensions 100 × 25 mm (Figure 2) [43].

Internal bond (IB) was measured following EN 319:2005 with the specimen dimensions 50 × 50 mm [44]. All mechanical properties (TS, SS, IB, MOE, MOR, and SWR) were determined using a Zwick/Roell 250 8497.04.00 test device (Ulm, Germany) under constant climatic conditions (rel. humidity 65%, ambient temperature 20 °C). Light microscopy was carried out with a Nikon Z1500 stereomicroscope, equipped with a Nikon Eclipse 50i (Tokyo, Japan) under a magnification of 10× and the software NIS-Elements Analytics D.5.20.02 (Tokyo, Japan). Scanning electron microscope (SEM) images were conducted by using a Zeiss Ultra Plus field emission scanning electron microscope (Oberkochen, Germany) following the procedure according to Sepperer et al. (2021) [45].

For statistical evaluation, IBM SPSS was used for descriptive data exploration applying univariate and multivariate methods. To determine differences between the reference and the reinforced specimens, an ANOVA, at a significance level of 95%, was used. For differences within the reinforced variants, a multivariate ANOVA was applied to determine the influence of “Type of reinforcement” (cellulose A and cellulose B, and flax C) and “adhesive amount” (A+, B+, and C+) with the “density” as the covariant. The explanatory power of the variables was evaluated by the determination of partial eta-squared values (ƞ^2^) in ANOVA. The significance of correlations (Pearson) was evaluated using two-sided confidence intervals of 95%.

## 3. Results & Discussion

### 3.1. Density and Thickness

The density is one of the major factors influencing physical and mechanical properties of wood-based materials. The modulus of elasticity, tensile- and compressive strength increases with higher densities for laminar structured boards [46].

The mean density of variant A plywood accounts for 0.874 (SD = 0.034) g/cm^3^ and is 2.70% higher compared to the reference sample with a density of 0.851 (SD = 0.024) g/cm^3^. Applying a higher resin content in the wood-fabric glue line in variant A+ plywood results in 3.64% higher density than the reference. Variant B density mean increased by 3.17% and the mean density of variant B+ by 4.58%. Variant C density mean decreased by 2.47% and 4.47% for the mean density of C+.

A significant influence of the density between reinforcement (A/A+, B/B+, and C/C+) and the non-reinforced reference (Ref) is not given (*p*-value 0.906; ƞ^2^ 0.000). A trend of increasing density for reinforced specimen compared to the reference is detected.

**Figure 3:**
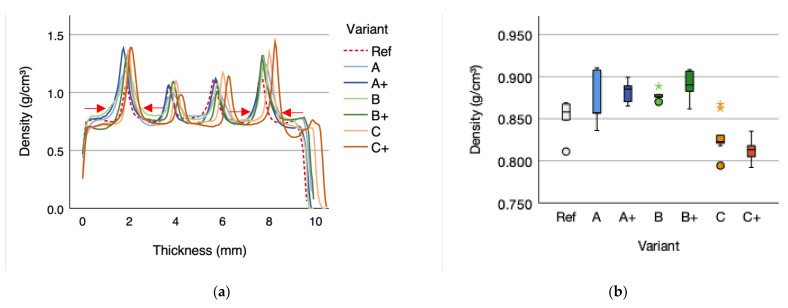
(**a**) Density profile and (**b**) density of the different variants of five-layered 10-mm plywood with reinforcement (A/A+, B/B+, and C/C+). The outliers are marked by “*” and “°”.

Density differences (Figure 3b) between A/A+ and B/B+ can be explained by the additional glue lines (Figure 1) and the higher amount of adhesive for A+ and B+ as compared to the reference, A and B. The adhesive amount for variant A+, B+, and C+ specimens was 40 g/m^2^ (density1.325 g/cm³) higher for bonding the cellulose and flax fabric (50 g/m² per layer) to the singular plies compared to the variant A and B specimens This effect could not be stated for the flax fiber reinforced specimen C and C+, displaying a negative trend.

Concluding, that variances in singular ply densities are greater than influences caused by additional reinforcing cellulose and flax fiber fabric and the additional adhesive amount. The density for beech wood (*Fagus sylvatica* L.) at zero moisture content (MC_0_) according to Lohmann (2010) ranges between 0.490 and 0.680 to 0.880 g/cm³ and for the moisture content of 12% (MC_12_) between 0.540 and 0.910 g/cm³ [36]. For plywood the density range is 0.760 to 0.810 g/cm³ [47]. According to Mahút and Réh (2007), the density of plywood is 10–15% higher compared to the used native wood species raw material density due to influences caused by pressing, temperature, and the adhesive [2]. To estimate the effect of density variances, pre-measurement of each plie before adhesive application should be considered.

The density profile (Figure 3a) is typical for laminar structured wood-based composite products such as plywood or laminated veneer lumber (LVL) [48]. Density peaks correspond to the glue line due to the higher density of the adhesive [49]. Changes are detected regarding the density distribution within the glue line as well as changes in the glue line thickness. Notables are the density peaks between the two outer glue lines (higher) of the reinforced samples compared to the two inner glue lines (lower) due to the fiber reinforcement and additional adhesive amount. The effect for A+, B+, and C+ could be due to the higher adhesive amount, increasing the density. Thickness differences of the glue line are notable for A/A+ and C/C+ compared to B/B+. Variant B/B+ displays a wider density distribution within the glue line, indicating a better adhesive distribution. Variant A/A+ and C/C+ have sharper curvature within the glue line (Figure 3a): red arrows). Both variants had similar failure behavior of internal bond and shear strength. Indicating that the hydrophobic pretreatment of variant B/B+ improves the bonding performances between veneer plies and the cellulose fabric.

**Figure 4:**
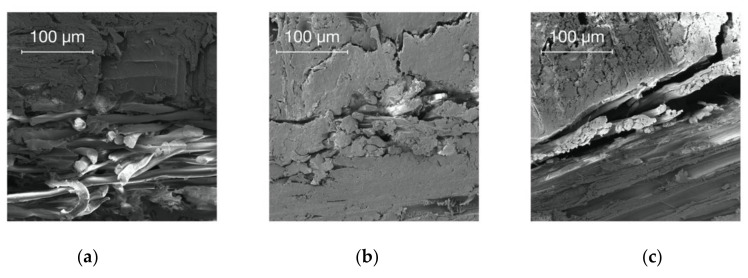
(**a**) SEM image of cellulose fabric A, (**b**) SEM image of cellulose fabric B, and (**c**) SEM image of flax fiber.

This is supported by the reflected-light microscope images, displaying the impact of cellulose fiber reinforcement within the glue line for the cellulose fabric A and B. The fabric B was within the glue line harder detectable than fabric A. The influence of adhesive penetration into the cellulose and flax fabrics and the surrounding cell wall structure is hardly detectable due to the colorless nature of urea-formaldehyde [50]. To overcome this disadvantage, Gößwald et al. (2021) suggested the use of pigments added to the UF as colorant to improve the visibility of UF distribution within lightweight particleboards [51]. Xing et al. (2004) used 0.01% toluidine blue O (TBO) solution to generate a fluorescence of UF under light microscope [52]. To clarify the hypothesis scanning electron microscopy (SEM) imaging of the cellulose fiber adhesive matrix was conducted. Kawalerczyk et al. (2021) used SEM to investigate the surface structure of UF wood adhesive filled with cellulose nanofibers [53]. Based on the SEM images, the differences between the different variants are clarified. The fabric of A/A+ is clearly visible within the glue line (Figure 4a), with some gradation the same applies to C/C+ (Figure 4c). In contrast, B/B+ is hardly detectable within the glue line indicating an improved adhesive penetration within the fabric (Figure 4b).

For the plywood board thickness (Table 2) no influence of the fiber reinforcement between the reference and the reinforced specimens does exist (*p*-value 0.409; ƞ² 0.000). Reinforced samples tend to slightly increase in thickness compared to the reference mean.

In contrast, the “Type of reinforcement” (*p*-value 0.001; R² 0.916) does significant influence the thickness of reinforced specimen. The differences between A (untreated cellulose) and B (hydrophobic treated) are not significant. Flax (C) tends to increase and differs significantly from fabric A (*p*-value 0.001) and B (*p*-value 0.001). The factor “Adhesive amount” (*p*-value 0.338; F 0.957) is insignificant but tends to increase thickness with higher amounts. Similar effects are given for the “Degree of compression” due to its dependence on the thickness.

The correlation between density and thickness is significant (R² 0.647; *p*-value 0.001). This is in accordance with the findings of Kallakas et al. (2020), stating the fact of higher density with lower thickness [54]. The compression degree ranges between 5% for C+ and 11% for B. This is within the range according to Bekhta et al. (2009) for plywood [55]. The veneer thickness does have a distinct influence on modulus of rupture and tensile strength for laminar wood-based products. The strength of laminar structures declines with increasing singular plie thickness. The compression of veneer does significant influence the modulus of elasticity and tensile strength [46].

### 3.2. Modulus of Elasticity & Modulus of Rupture

**Figure 5:**
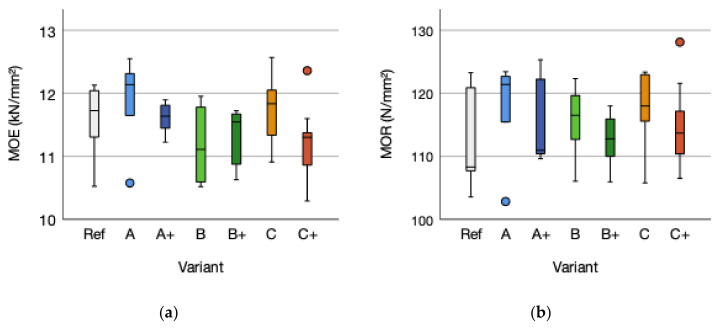
(**a**) Modulus of elasticity (MOE) and (**b**) modulus of rupture (MOR) of the reference (Ref) and the reinforced variants A, B, and C and the variants with higher adhesive amount A+, B+, and C+. The outliers are marked by “°”.

Variant A modulus of elasticity mean of 11,845 (SD = 782) N/mm^2^ increased by 2.59% compared to the reference modulus of elasticity mean of 11,546 (SD = 655) N/mm^2^. The modulus of elasticity means for A+ increased by 0.49%. In contrast, variant B and B+ modulus of elasticity mean decreased by 3.07%, respectively 2.22%. The modulus of elasticity means for C increased by 1.63% and the variant C+ decreased by 2.71% (Figure 5a).

The modulus of rupture means of variant A with 117.16 (SD = 8.59) N/mm² and A+ (115.71 (SD = 7.46) N/mm² increased by 3.91% and 2.63%, respectively, compared to the reference mean of 112.75 (SD = 8.75) N/mm². The mean of variant B increased by 2.39% while variant B+ decreased by 0.19%. The flax fiber reinforced variant C mean increased by 4.83% and by 1.91% for C+ mean (Figure 5b).

The reinforcement does not influence the modulus of elasticity (*p*-value 0.826; ƞ² 0.001) nor modulus of rupture (*p*-value 0.333; ƞ² 0.023) compared to the reference. The reinforcement tends to influence the modulus of elasticity in a negative way, but to improve the modulus of rupture performance. The adhesive amount does not influence the modulus of elasticity (*p*-value 0.641 F 0.224) and modulus of rupture (*p*-value 0.380 F 0.802). The reinforcement fabric does not influence the modulus of rupture (*p*-value 0.030 F 4.108) and modulus of elasticity (*p*-value 0.006 F 6.584) of the plywood structure.

Within the groups, non-significant trends could be stated regarding the effect of adhesive amount on modulus of elasticity. For the non-treated cellulose variant, A and flax fiber reinforcement C, a negative trend for increasing adhesive amount is detected. In contrast, variant B displays a positive effect of higher adhesive amount on modulus of elasticity. This cannot be stated for the modulus o rupture. For A/A+, B/B+s and C/C+, a trend of negative influence of higher adhesive amount on the modulus of rupture is detected. The means of the reinforcement type displays higher performance for non-treated cellulose (A/A+) and flax fiber (C/C+) compared to the reference mean and pretreated cellulose (B/B+). The trend of modulus of rupture and modulus of elasticity is in accordance with Kawalerczyk et al. (2021), stating that the adhesive amount can be reduced to 120 g/m² without significantly influencing the mechanical properties [53].

The stress–strain diagram, based on the mean curve for each variant and obtained from the three-point bending test under loading, display no improvement regarding fiber-reinforcement within the linear elastic region. This changes subsequently at the non-linear elastic region where the fiber-reinforcement surpasses the reference. Within the elastic-plastic region the fiber-reinforced variants are capable to withstand failure due to fracture by stress transfer within the reinforcing layer based on the higher tensile strength properties of the fiber fabric. Notable is the difference of variant A/A+ und C/C+ regarding the adhesive amount. Lower adhesive amount, as by A and C, improve the behavior within the non-linear elastic region. This is vice-versa for hydrophobic pretreated variant B/B+. The findings are underlined by the failure pattern. All test specimens failed at the tension side. The variants A/A+ and C/C+ show longer crack-length along the outer glue line at the tension side. This indicates a failure behavior due to stress transfer within the fabric.

**Figure 6:**
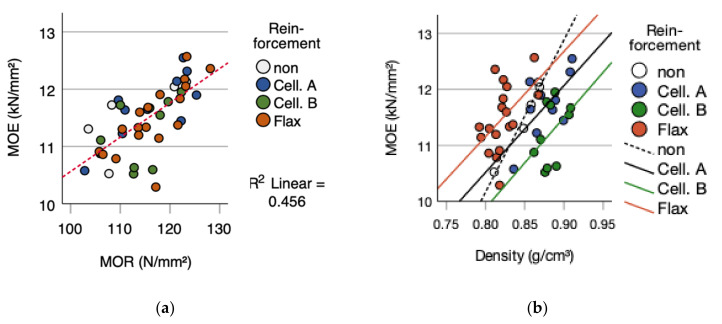
(**a**) MOE versus MOR and (**b**) MOE versus density.

The correlation between density and modulus of elasticity is not given with R^2^ = 0.093 and a *p*-value of 0.047. Within the different fabrics a divergent picture is displayed. The non-reinforced reference (R² = 0.968; *p*-value = 0.002), Cellulose fabric A (R² = 0.454; *p*-value = 0.033) and the flax fiber reinforcement (R² = 0.25; *p*-value = 0.035) do have a significant correlation between density and modulus of elasticity. Cellulose fabric B does not a correlation, due to R² is 0.187 and a *p*-value of 0.211 (Figure 6b). The correlation between modulus of elasticity versus modulus of rupture with an R^2^ = 0.456 (*p*-value = 0.001) (Figure 6a) is slightly given. The non-influence of density on the modulus of elasticity is not according to Kollmann (1955) and Wagenführ/Scholz (2008) [10,56]. The correlation between the degree of compression and modulus of rupture, as stated by Niemz (1993) [46], is not detected (R² < 0.001; *p*-value 0.984).

### 3.3. Tensile Strength

**Figure 7:**
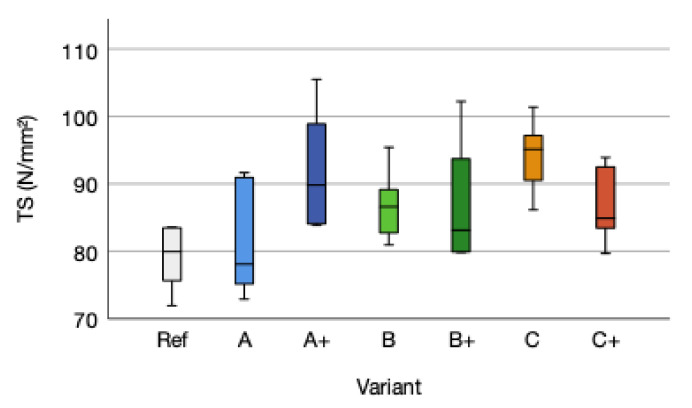
Tensile strength (TS) distribution of the reference (Ref) and the reinforced variants A, B, and C and the higher adhesive amount variants A+, B+, and C+.

Tensile strength is considered to be an important factor for plywood applications prone to load bearing structures [57]. Tensile strength means of variant A with 81.76 (SD = 8.91) N/mm^2^ increased by 2.84 N/mm^2^ (+4%) compared to the reference TS mean of 92.43 (SD = 5.06) N/mm^2^. The A+ variant tensile strength mean increased by +18%. The tensile strength means of variant B increased by 10%, respectively by 11% for variant B+. Variant C increased by 19% and C+ by 10% (Figure 7).

No influence of the fiber reinforcement (Type A, B, and C) on the tensile strength compared to the reference can be stated (*p*-value 0.020; ƞ² 0.154). A general tendency of fiber reinforcement improving the tensile strength is detected. Between the fiber reinforcement variants, a higher adhesive amount does not influence the tensile strength (*p*-value 0.753; F 0.101) as well as the type of fiber reinforcement (*p*-value 0.973; F 0.028). A positive trend for increasing tensile strength due to higher adhesive amount is noted. Within the cellulose A/A+ and B/B+ the tensile strength mean increased due to higher adhesive amount. This is not detected for flax fiber reinforcement C/C+. The effect of urea-formaldehyde (UF) with an adhesive amount of 200 g/m² and a lower increase in tensile strength for flax is notable, if compared to Jorda et al. (2021) [27]. No correlation between density and tensile strength is given due to a R^2^ of 0.019 and a *p*-value of −0.137. This is not in accordance with Niemz (1993). The tensile strength should increase with higher density [46]. Additionally, no correlation for tensile strength versus modulus of elasticity due to R² = 0.005 and a *p*-value of −0.067, tensile strength versus modulus of rupture (R² = 0.003; *p*-value 0.056) and tensile strength versus shear strength (R² = 0.004; *p*-value −0.028) and tensile strength versus compression (R² = 0.012; *p*-value 0.538) could be detected.

### 3.4. Shear Strength and Internal Bond

**Figure 8:**
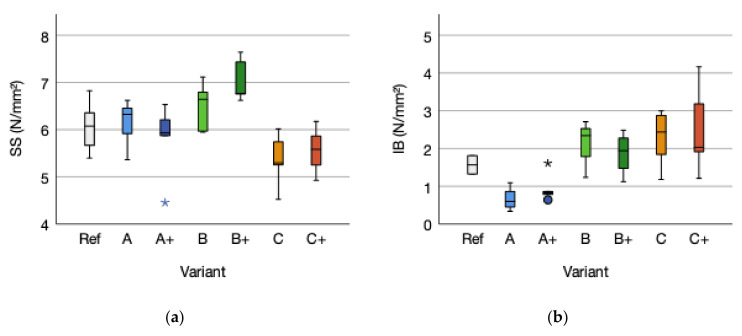
(**a**) Shear strength (SS) and (**b**) Internal bond (IB) distribution of the reference (Ref) and the reinforced variants A, B, and C, and the higher adhesive amount variants A+, B+, and C+. The outliers are marked by “*” and “°”.

The performance of plywood is strongly depended on the bonding performance of the adhesive used for joining the singular veneer plies [58]. Reinforcement does not have a significant influence on shear strength (*p*-value 0.721; ƞ² 0.003) and internal bond (*p*-value 0.666; ƞ² 0.006). Shear strength tends to decrease with reinforcement whereas internal bond tends to increase for reinforced specimens. The differences between the types of reinforcement are significant for internal bond (*p*-value 0.001; R² 0.550). Cellulose A displays the lowest internal bond, followed by cellulose B and flax with the highest. The adhesive amount tends to insignificant increase the internal bond (*p*-value 0.835). Shear strength is not influenced by the type of reinforcement (*p*-value 0.011) nor the adhesive amount (*p*-value 0.128). The trend given for the internal bond regarding the reinforcement is not stated for shear strength due to the given order flax (lowest shear strength), cellulose A, and cellulose B (highest shear strength). Higher adhesive amounts tend to increase shear strength. The shear strength means of variant A with 6.14 (SD = 0.51) N/mm^2^ equaled by 0.07 N/mm^2^ (+0.01%) compared to the reference SS mean of 6.07 (SD = 0.56) N/mm^2^. The shear strength means for A+ decreased by 4.45%. In contrast, the shear strength means of variant B and for variant B+ increased by +6.92%, respectively by 15.98% compared to the reference mean. Flax fiber reinforced variant C lowered by 11.86% and C+ by 8.24% (Figure 8a).

Differences are given regarding the fracture pattern. Type A and A+ displayed a failure within the fabric embedded to the glue line 1. In contrast, type B and B+ failed mainly within the glue line 2. A fracture within glue line 1 was only detected for two specimens of variant B. Variant C and C+ failed subsequently within the wooden surface layer. Indicating influences of the different fabrics on the shear strength as confirmed by the trend of the different reinforcing fabrics. The internal bond means of variant A with 0.66 (SD = 0.32) N/mm^2^ decreased by 0.91 N/mm^2^ (−57.96%) compared to the reference internal bond mean of 1.57 (SD = 0.35) N/mm^2^. The internal bond means for A+ decreased by −39.49%. In contrast, the internal bond means of variant B and variant B+ increased by 33.76%, respectively by 19.75% compared to the reference mean. Differences are given regarding the type of fracture. Variant A and A+ displayed a failure within the fabric. In contrast, variant B and B+ failed with a combination of wood/fabric fracture. The variant C failed primarily within the fabric and C+ displays a tendency within the fabric. The different failure pattern detected by the visual analysis are confirmed with significant influence of the fabric on the internal bond (*p*-value 0.001; R² 0.550) (Figure 8b). Internal bond according to EN 319 is designed for testing wood particle-based materials. It is considered to be a simple test [59] and can be used to evaluate the perpendicular tensile strength of plywood [60]. The usability for reinforced plywood due to less pre-test preparation compared to EN 314 was evaluated. No correlation between shear strength and internal bond could be stated (R² 0.033; *p*-value 0.286). The comparison of the fracture pattern of shear strength and internal bond reveled similarities of the failure mode to a certain extent, but was not conclusive. Influence on the test specimen during preparation and testing must be mentioned. First, the influence of temperature (>200 °C) during gluing of the samples to the aluminum metal block causing stress within the surface plies due to sharp temperature differences. Second, adhesive overlap on the outside of the specimen in tensile direction. This are two potential biases for the test results, as mentioned, beside other factors, by Rathke et al. (2012). To improve and for a deeper understanding of the strain distribution under tensile loading, the use of digital image correlation (DIC) is recommended for the analysis, as a value adding method according to Li et al. (2020) [58].

### 3.5. Screw Withdrawal Resistance

**Figure 9:**
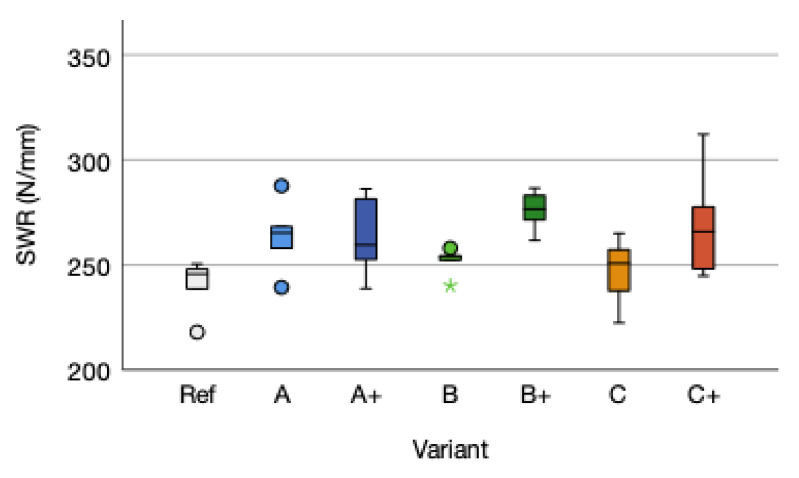
Screw withdrawal resistance (SWR) distribution according to EN 320 of the reference (Ref) and the reinforced variants A, B, and C, and the higher adhesive amount variants A+, B+, and C+. The outliers are marked by “*” and “°”.

The main connecting element for structural wood-based materials, such as plywood or oriented strand board (OSB) are screws. Therefore, the screw withdrawal resistance is one of the important factor for wood-based materials used for construction applications [61]. The screw withdrawal resistance means of variant A with 263.70 (SD = 17.49) N/mm improved by 23.52 N/mm (+9.79%) compared to the reference mean of 240.18 (SD = 13.17). Variant A+ increased by 9.80%. The screw withdrawal resistance means of B and B+ increased by 4.81%, respectively by 14.87% compared to the screw withdrawal resistance reference mean. Variant C improved by 2.99% and the variant C+ by 4.92% (Figure 9).

The screw withdrawal resistance is not influenced by the reinforcement due to a *p*-value of 0.257 (ƞ² 0.188). A tendency for improving the screw withdrawal resistance is given. Neither adhesive amount (*p*-value 0.017; F = 6.731) nor the type of fiber reinforcement (*p*-value 0.951; F = 0.050) does significantly influence the screw withdrawal resistance. Higher adhesive amount tends to increase the screw withdrawal resistance. The type of reinforcement displays a slight improvement for flax fabric compared to the cellulose A and B. Comparing the trend within the different fabrics a non-significant tendency of higher adhesive amount improving the screw withdrawal resistance can be detected. The pretreatment of the cellulose fabric influences the screw withdrawal resistance performance. Additional adhesive amount is increasing density. The correlation between density and screw withdrawal is given by R^2^ = 0.241 and a *p*-value of 0.013. This is in accordance to the general assumption that density is influencing the screw withdrawal resistance [10,62] beside other wood related parameters (fiber direction, species, moisture content, and temperature) [63]. Fiber reinforcement does influence the screw withdrawal resistance with regard to the position of the reinforcing fabric within the board structure [62,63,64,65,66]. The effects of the fabric singular characteristics should be further considered for further research [27].

## 4. Conclusions

**Figure 10:**
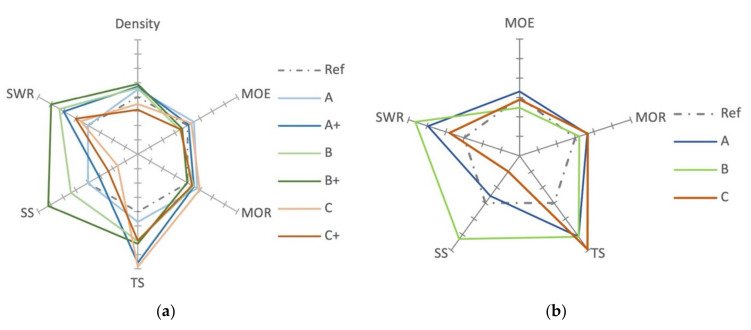
(**a**) Percentage-based performance of the variants (axis interval 5%). (**b**) Intermediate percentage-based performance of cellulose A, B, and flax C (axis interval 5%).

The aim of the study was to determine the influence of unidirectional fabrics of: (a) non-treated cellulose (A), (b) hydrophobic pretreated cellulose fabric (B), and (c) flax fiber (C), with two different adhesive amounts of urea-formaldehyde on the mechanical properties of five-layered beech plywood (~10 mm). The percentage-based performance (Figure 10a,b axis interval 5%) reveal the varying influence of different fabrics on the mechanical properties’ performance. The behavior of cellulose fabric A is comparable to the flax fiber fabric. This is due to the higher cellulose content of flax and a similar hydrophilic behavior, as well as the bonding performance of urea-formaldehyde. Hydrophobic pretreatment improved the tensile strength (10−11%), shear strength (7−16%), screw withdrawal resistance (11−15%), and modulus of rupture (2−0%), but lowered modulus of elasticity (3−2%) of the cellulose fabric B compared to the reference (Ref).

It must be stated that most of the effects are statistically insignificant. This is due to the low areal weight (50 g/m²) of the reinforcing nonwovens used in the study as well as the position of the reinforcement within the outer glue line of the plywood. This hinders the reinforcing fabrics from exploiting its full potential. The use of unidirectional reinforcement within a plywood structure has to be questioned and seems to be more applicable to unidirectional laminar structured veneer-based products such as laminated veneer lumber (LVL). The usability of internal bond determination according to EN 319 for nonwoven natural fiber reinforced plywood must be neglected.

This research did not account for press-parameters and different adhesive types. Further research should focus on the bonding quality of the nonwovens (adhesive system/cohesion and adhesive-nonwoven penetration), interfacial interactions in the adhesive nonwovens matrix and alternatives of more ecological friendly adhesive systems as well as the effect of higher areal weights of the nonwovens. In addition, the effect of reducing adhesive amount on the mechanical properties, as stated by Kawalerczyk et al. (2021), should be taken into account for further research [53].

## Figures and Tables

**Table 1:** Design of experiments for the mechanical properties of unidirectional cellulose and flax fiber reinforced plywood.

Variant	Fiber Reinforcement	Adhesive Application		Density	MOE	MOR	TS	SS	IB	SWR
		Wood/Wood	Wood/Fabric	Number of Specimen (N)						
		(g/m²)								
Ref	-	160	-	5	5	5	5	5	5	5
A	Lyocell A	160	160	5	5	5	5	5	5	5
A+	Lyocell A	160	200	5	5	5	5	5	5	5
B	Lyocell B	160	160	5	5	5	5	5	5	5
B+	Lyocell B	160	200	5	5	5	5	5	5	5
C	Flaxtape	160	160	9	9	9	5	9	9	9
C+	Flaxtape	160	200	9	9	9	5	9	9	9

**Table 2:** Thickness of five layered plywood with reinforcement (A/A+, B/B+, and C/C+).

Variant	Reinforcement	N	Thickness (mm)				Compression Degree (%)
			Min	Mean	Max	SD	
Ref	-	5	9.98	10.02	10.05	0.03	8.91
A	A	5	9.91	9.96	10.03	0.06	9.45
A+		5	9.80	9.83	9.87	0.03	10.64
B	B	5	9.73	9.78	9.84	0.05	11.09
B+		5	9.87	9.96	10.05	0.07	9.45
C	C	9	10.18	10.34	10.61	0.16	6.00
C+		9	10.32	10.45	10.67	0.11	5.00

## Data Availability

Not applicable.
